# Effects of Traditional Versus Horizontal Inertial Flywheel Power Training on Common Sport-Related Tasks

**DOI:** 10.1515/hukin-2015-0071

**Published:** 2015-10-14

**Authors:** Moisés de Hoyo, Borja Sañudo, Luis Carrasco, Sergio Domínguez-Cobo, Jesús Mateo-Cortes, María Monserrat Cadenas-Sánchez, Sophia Nimphius

**Affiliations:** 1 Fitness Section. Sevilla Football Club. Seville, Spain.; 2 Department of Physical Education and Sport. University of Seville. Seville, Spain.; 3 School of Exercise and Health Sciences. Edith Cowan University. Joondalup, Australia.

**Keywords:** maximal power output, half squat exercise, front step exercise, performance

## Abstract

This study aimed to analyze the effects of power training using traditional vertical resistance exercises versus direction specific horizontal inertial flywheel training on performance in common sport-related tasks. Twenty-three healthy and physically active males (age: 22.29 ± 2.45 years) volunteered to participate in this study. Participants were allocated into either the traditional training (TT) group where the half squat exercise on a smith machine was applied or the horizontal flywheel training (HFT) group performing the front step exercise with an inertial flywheel. Training volume and intensity were matched between groups by repetitions (5–8 sets with 8 repetitions) and relative intensity (the load that maximized power (Pmax)) over the period of six weeks. Speed (10 m and 20 m), countermovement jump height (CMJH), 20 m change of direction ability (COD) and strength during a maximal voluntary isometric contraction (MVIC) were assessed before and after the training program. The differences between groups and by time were assessed using a two-way analysis of variance with repeated measures, followed by paired t-tests. A significant group by time interaction (p=0.004) was found in the TT group demonstrating a significantly higher CMJH. Within-group analysis revealed statistically significant improvements in a 10 m sprint (TT: −0.17 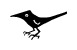 0.27 s vs. HFT: −0.11 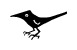 0.10 s), CMJH (TT: 4.92 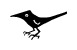 2.58 cm vs. HFT: 1.55 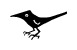 2.44 cm) and MVIC (TT: 62.87 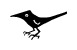 79.71 N vs. HFT: 106.56 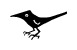 121.63 N) in both groups (p < 0.05). However, significant differences only occurred in the 20 m sprint time in the TT group (−0.04 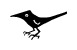 0.12 s; p = 0.04). In conclusion, the results suggest that TT at the maximal peak power load is more effective than HFT for counter movement jump height while both TT and HFT elicited significant improvements in 10 m sprint performance while only TT significantly improved 20 m sprint performance.

## Introduction

The generation of force over a short period of time is required in many sports activities (Cormie et al., 2007). Within them, movements requiring rapid development of force include sprint acceleration, jumping and change of direction (COD) (Newton and Kraemer, 1994). According to the Newton’s second law [F = m·(Δv/t)], increased force development will result in an increased velocity of movement (Newton et al., 2012). Therefore, researchers have focused on importance of the measure of power for performance as it describes the interaction of both force and velocity (P = F*v) (Baker, 2001a; Baker, 2001b; Haff et al., 2001; Kawamori et al., 2005). Consequently, improvements in maximal power output have induced an enhanced performance in jumping, sprinting, and COD tests, as well as beneficial changes in isometric strength (McBride et al., 2002; Winchester et al., 2005). However, it should be noted that changes in power output have not always been shown to have significant relationships with improvements in performance as measured by sprint speed or vertical jump ability (Harris et al., 2008b; Nimphius et al., 2010). The reason for this inconsistency in the relationship between power and sports activities is currently unknown, but some authors have proposed the specificity of training may influence the transfer to performance (Young, 2006). This lack of specificity may explain the mixed findings on the association between improved power output and enhanced performance. However, there are other variables that may influence the relationship between improved power output and enhanced performance such as the difficulty of identifying the training load to use for maximizing power or the length of training intervention.

Many interventions for power development include lower-body exercises involving the triple extension of the knee, ankle, and hip that avoid a deceleration phase, as they are considered closest to the actions of sprinting and jumping performed in many sports (Kawamori and Haff, 2004). Therefore, common exercises for power development are ballistic exercises (loaded jump squats), plyometrics (jumps and bounds) and Olympic lifts (e.g. snatch and clean). However, a majority of these exercises focus on movement in the vertical plane despite most athletic pursuits requiring horizontal movement (e.g. sprinting and changing direction). As previously mentioned, this lack of specificity has been cited as the potential reason for a lack of transfer of power training to common sport-related tasks (Young, 2006). To improve specificity, training in the horizontal plane using a flywheel apparatus allowing for resistance in this proposed “specific plane” while providing a unique net joint impulse at the knee and hip was proposed (Chiu and Salem, 2006).

Although specificity may influence the effectiveness of power training on transfer to sport activities, strength and conditioning literature considers the production of peak power output as a cornerstone of athletes’ performance (Turner et al., 2012). As a result, a number of researchers and practitioners have suggested that training at loads where mechanical power output is maximized (Pmax) is optimal for improvements in athletic performance (Cormie et al., 2007; Harris et al., 2008a; Sleivert and Taingahue, 2004; Zink et al., 2006). However, the research states an enormous range of percentages (30–80%) for the load that maximize power (Cormie et al., 2011; Harris et al., 2008a; Hopkins, 2005; Jones et al., 2009; Moss et al., 1997; Wilson et al., 1993) depending on the athlete training history, exercise type and strength level of the athlete (Harris et al., 2008b; Newton and Dugan, 2002; Sleivert and Taingahue, 2004; Stone et al., 2003). Therefore, it would seem important to specifically identify the load where Pmax occurs for each individual participant on specific exercises to adequately investigate the effects of different modalities of training at Pmax on force, power, and functional performance (Harris et al., 2008a).

Previous research (Newton et al., 2006) has demonstrated that four weeks of resistance training using traditional vertical exercises (e.g. jump squats on a smith machine) performed at a Pmax resulted in an attenuation of the decrement in jump performance during a competitive season in female volleyball players. Furthermore, Harris et al. (2008a) found that seven weeks of either heavy squat training or squat training at the Pmax load were effective to improve the 10 and 30 m sprint performances of well-trained rugby league players. However, there was no association between the change in power output and change in sprint performance. Despite the lack of association between the change in power output and change in sprint time, short-term training at Pmax (between four to seven weeks) did elicit improvement in common sport-related tasks and could be useful for a certain period of competition for athletes.

Specificity and training at a load that maximizes power output for a short period of time have been proposed as potential factors that contribute to improving the ability for increased power output to more effectively transfer to improved sport activities. As an alternative to these traditional and ballistic training methods, flywheel inertial devices have appeared increasingly in scientific research and are being incorporated into regular training programs (Chiu and Salem, 2006). The benefits of this device include eliciting a greater overall amount of muscle activity than traditional overload exercises (Norrbrand et al., 2010) and the ability to freely move in the three dimensions for a “more specific” training stimulus (Young, 2006; Lohnes et al., 2007). Therefore, this study aimed to compare the effects of six weeks of training at the individual Pmax load with a traditional half squat exercise (TT) versus a “more specific” front step exercise resisted in the horizontal plane by a flywheel device (HFT) on common sport-related tasks in physically active men. Based on the theory of specificity, we hypothesized that the two training modalities would elicit significantly different changes in sprint speed, change of direction ability (COD), countermovement jump height (CMJH) and maximal voluntary isometric strength (MVIC).

## Material and Methods

### Participants

Thirty-two healthy and physically active males (mean ± SD; age: 22 ± 2 years, body height: 176.98 ± 7.52 cm, body mass: 76.92 ± 3.72 kg, BMI: 24.55 ± 2.20 kg·m^−2^) volunteered for this study. They were identified as active according to the minimal activity guidelines released by the American College of Sports Medicine (Garber et al., 2011) as they reported more than thirty minutes of moderate physical activity five times a week as estimated by the International Questionnaire of Physical Activity (IPAQ) (Craig et al., 2003). However, individuals already participating in resistance training within the last three months were excluded from the research (n = 9). All testing procedures and the training protocol were explained and participants gave written informed consent prior to the commencement of the study. The University of Seville Research Ethical Committee approved the experimental protocol and the procedures involved.

### Procedures

**Figure 1:**
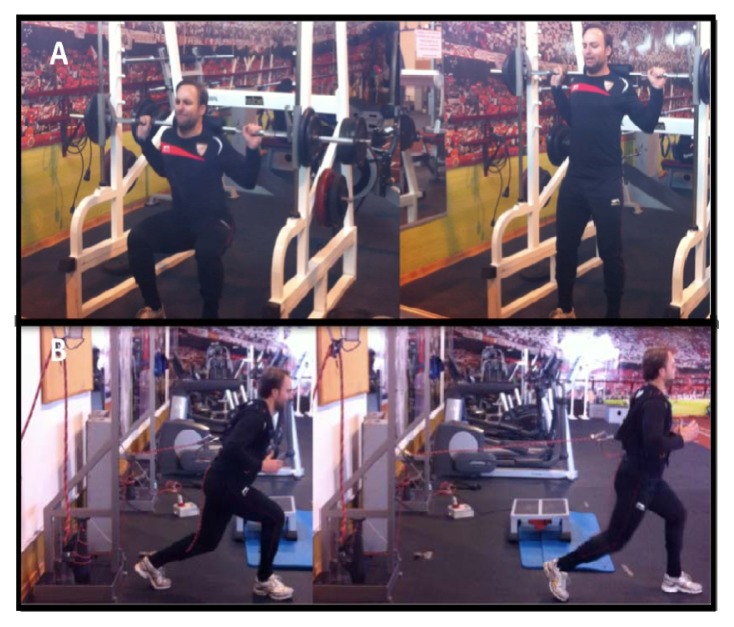
A. Traditional training using a half squat on a smith machine; B. horizontal flywheel training using a front step with an inertial flywheel device

**Figure 2:**
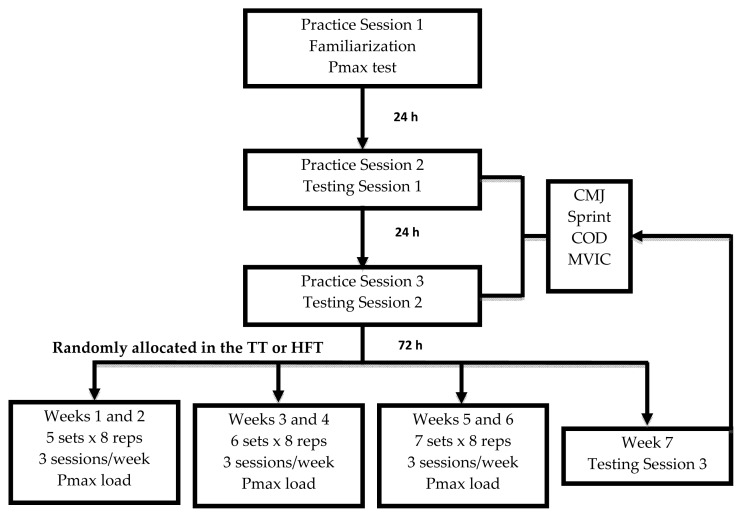
Experimental timeline. Pmax = maximum peak power output; CMJ = countermovement jump test; Sprint = 10 and 20 meter sprint tests; COD: 20 m change of direction test; MVIC = maximal voluntary isometric contraction test; reps = repetitions

To compare the effect of six weeks of training at loads that elicit maximum power (Pmax) using two different strength training programs, participants were randomly allocated to either the: 1) traditional training (TT) group (n=12) with the half squat exercise on a smith machine (Figure 1-A) or 2) specific training (HFT) group (n=11) with a front step exercise using an inertial flywheel (Figure 1-B). Table 1 shows the descriptive characteristics of both groups and demonstrates there was no significant difference between the two groups (p > 0.05). Each participant visited the laboratory and completed a familiarization session and two testing sessions separated by at least 24 hours. During the familiarization session, a full explanation of the experimental protocol was given to the participants and they were permitted to practice all the tests. In addition, the individual Pmax load of both exercises (half squat and front step) was determined for all participants. To assess reliability, participants performed two testing sessions to assess CMJH, 10 and 20 m sprint time, COD ability and MVIC. Tests were separated by 3 min rest periods. Three trials of each test were permitted with the best score in each test being used for subsequent analysis. Reliability of measures was assessed using intraclass correlations (ICC) and the coefficient of variation (CV) and demonstrated high reliability for all measures: ICC (0.90–0.96) and CV (2.7–5.2%). For training, all participants performed three exercise sessions a week (Monday, Wednesday and Friday) for six weeks. Each session consisted of a standardized warm-up of 5 min on a cycle ergometer (Ergoline 900, Ergometrics, Bitz, Germany) at 80 Watts and 80 RPM. Strength training involved an increasing volume program (numbers of sets performed) at the same relative intensity for both groups (load where Pmax occurred during the initial assessment). During the first week, participants performed 5 sets of 8 repetitions and the volume increased one set every two weeks (e.g. 6 sets for weeks three and four, 7 sets for weeks five and six) keeping the number of repetitions fixed. Participants were instructed to execute each repetition at maximal velocity. In order to achieve the same training volume by repetitions, HFT participants performed 4 repetitions with each leg. Figure 2 shows the experimental timeline.

### Measures

#### Peak power determination in the half squat

Participants were positioned in a half-squat position (relative knee flexion of 90°) in a smith machine (FITLAND, Seville, Spain) and were instructed to extend the legs fully (considered 0° or full extension). The concentric phase was performed by instructing the participant to move the bar as quickly as possible back to a standing position in an attempt to maximize power output (Blazevich et al., 2001; Newton et al., 1996). The participants’ feet did not leave the ground and the bar was not allowed to leave the participants’ shoulders. Each participant performed a protocol where the load was increased (after a rest period of 3 min) by 10 kg each repetition to determine the load at which Pmax was obtained. The test finished when there was a decrease in the power output as compared with the previous repetition. Power was measured using a linear transducer (ERGOTECH Consulting, Spain). This linear position transducer sampled at a frequency of 1000 Hz. The following derived mechanical variables were calculated by the software: displacement (m) was obtained by integration of velocity (m·s^−1^) data with respect to time; instantaneous acceleration (m·s^−2^) was obtained from differentiation of velocity with respect to time; instantaneous force (N) was calculated as F = m · (a + g), where m is the moving mass (kg) and g is the acceleration due to gravity; instantaneous power output (W) was calculated from the product of the instantaneous force and bar velocity (P = F · v). The load that maximized peak power for this group during the half squat exercise was 72.21 ± 12.54 kg.

#### Peak power determination in the front step

Participants were placed in a front step position and explosively performed a horizontal acceleration (or step). Each participant performed an incremental protocol using the flywheel inertial device (Sport Teach & Tools S.L.U, Spain). This device consists of a flywheel with two 1 kg masses positioned at opposite ends of a metal beam with a length of 0.46 m. A fixed axis is located at the center of the beam, about which the masses rotate. A cone is attached above the flywheel, and as the flywheel and cone spin, a tether winds and unwinds around the cone. The load was increased (after a rest period of 3 min) by 2 kg each repetition and determination of Pmax was considered complete when there was a decrease in the power output as compared with the previous repetition. As both legs were to be trained, the average of two loads at which Pmax occurred was used for subsequent training. The power output was measured using the previously described methods in the half squat but force was measured directly by a load cell (Model 333A, MuscleLab™, Ergotest AS, Langesund, Norway) connected to an A/D converter (MuscleLabTM, Ergotest AS, Langensund, Norway). The linear transducer and the load cell were placed in a horizontal position and attached to a harness on the participant. Instantaneous power output (W) was calculated from the product of the force (N) and velocity (m·s^−1^). The load that maximized peak power for this group during the front step exercise was 7.78 ± 4.22 kg.

#### 10 m and 20 m sprint tests

Sprint time was measured using dual beam electronic timing gates (OptoJump System; Microgate, Bolzano, Italy) at the distances of 10 and 20 m. The starting position was standardized with the left toe one meter back from the starting line and the right toe, in a staggered stance, approximately in line with the heel of the left foot. All assessments were performed on an indoor court surface, and participants wore rubber-soled track shoes. The participants performed three trials for each distance (10 and 20 m) with the best time used for subsequent analysis. A recovery time of 2 min between each attempt and 3 min between both distances was provided. The ICC and CV were 0.93 and 4.7% and 0.94 and 3.5% for the 20 m sprint, respectively.

#### Countermovement jump test

The countermovement jump (CMJ) was assessed using the OptoJump System (OptoJump System; Microgate, Bolzano, Italy). Three trials of the CMJ, with 60 seconds rest between trials, without arms (hands on hips) were performed. The countermovement phase included flexion to approximately 90° of relative knee flexion and then without pausing participants jumped upward as high as possible. To determine CMJH, elevation of the center of gravity (m) was calculated for all jumps by the equation: H=(t_v_^2^·g)/8; where H is the height and g is the gravitational acceleration (9.81 m·s^−2^) and flight time (t_v_) in seconds. The ICC and CV for CMJH was 0.96 and 2.7%, respectively.

#### 20 meter change of direction test

Participants performed three trials on a zigzag course consisting of four 5 m sections set out at 100° angles with a total distance of 20 m. This zigzag test was chosen as it required the acceleration, deceleration, and body control facets of change of direction, the familiarity of the participants with the test and the relative simplicity also meant that learning effects would be minimal (Little and Williams, 2005). All trials were performed on an indoor synthetic pitch, and electronic timing gates (OptoJump System; Microgate, Bolzano, Italy) were used to record completion times. The starting position was the same as reported in the sprint tests. A recovery time of 2 min between each attempt was allowed. The ICC and CV for the COD test was 0.90 and 4.4%, respectively.

#### Maximal voluntary isometric knee extension

The maximal isometric voluntary contraction (MVIC) during a leg extension was assessed using a load cell (Model 333A, MuscleLab™, Ergotest AS, Langesund, Norway) connected to an A/D converter (MuscleLabTM, Ergotest AS, Langensund, Norway) and sampled at 1000 Hz. Participants sat upright on a high-backed chair with the hips firmly secured and the knee positioned at 90° of flexion. The arms were folded across the chest while participants were asked to extend the knee with as much force as possible for 3 s. Three trials were completed and 2 min of rest were permitted between each trial of the MVIC. The ICC and CV for the MVIC were 0.95 and 5.2%, respectively.

### Statistical analyses

Mean and standard deviation (SD) were calculated for all variables. Statistical analysis was performed using SPSS 15.0 for Windows (SPSS Inc, Chicago, IL, USA). All variables were normally distributed as assessed by the Kolmogorov-Smirnov test. Variables were analyzed by a two-way analysis of variance with repeated measures: group (TT and HFT) and time (pre- and post-training) and follow-up comparisons were made with paired t-tests. Furthermore, a planned comparison analysis for the training variables (sprint speed, COD, CMJH and MVIC) within each group was assessed using paired t-tests. Statistical significance was set at p ≤ 0.05. The magnitude of effect (*d*) was calculated for paired variables (Cohen, 1998). The scale suggested by Rhea for magnitude of effect in strength training research was used to interpret the magnitude of effects in strength training: trivial (<0.35), small (0.35–0.80), moderate (0.80–1.50) or large (≥ 1.50) (Rhea, 2004).

## Results

### 10 m and 20 m sprint tests

**Figure 3:**
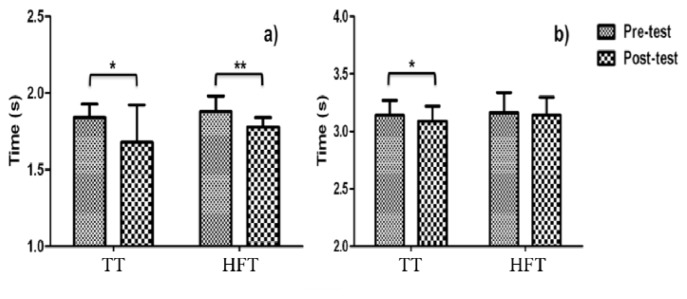
Pre- and Post-training (a) 10-m and (b) 20-m sprint times. TT = traditional training using a half squat on a smith machine; HFT = horizontal flywheel training using a front step with an inertial flywheel device. No significant group by time interaction (p > 0.05). * Significant planned comparisons within-group differences (p < 0.05). ** Significant planned comparisons within-group differences (p < 0.01)

No significant group by time interaction was observed for the 10 m and 20 m sprint tests. However, planned comparisons within group analysis revealed statistically significant changes from pre- to post-training for the 10 m sprint in both groups (TT: −0.17 ± 0.27 s, p = 0.05 vs. HFT: −0.11 ± 0.10 s, p = 0.01) with both TT and HFT participants showing a significant and moderate magnitude of effect change (TT: 0.91, moderate effect; HFT: 1.19, moderate effect) (Table 2). Planned comparisons of the 20 m sprint test revealed significant within group changes in the TT (−0.05 ± 0.6; p = 0.04), but not in the HFT group (−0.02 ± 0.05 s; p = 0.23). Figure 3 shows the 10 and 20 m sprint times before and after the intervention.

### Counter movement jump test

**Figure 4:**
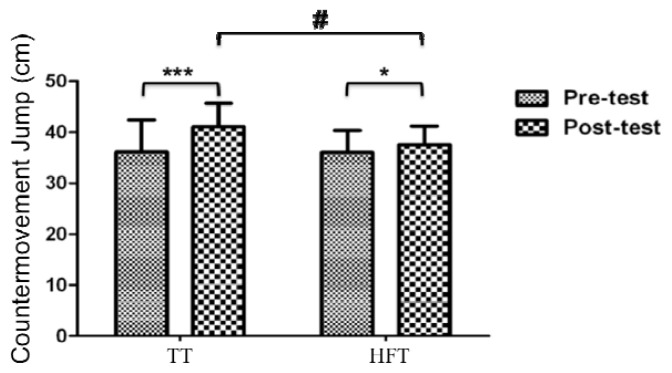
Pre- and post-training countermovement jump height (CMJH). TT = traditional training using a half squat on a smith machine; HFT = horizontal flywheel training using a front step with an inertial flywheel device. # Significant group by time interaction (p<0.05). * Significant within-group difference (p<0.05). *** Significant within group difference (p<0.001)

There was a significant group by time interaction in CMJ height (p=0.004). Within-group differences for the CMJ were observed in the TT (4.92 ± 2.58 cm; p = 0.001) and HFT group (1.55 ± 2.44 cm; p = 0.05) with a greater magnitude of effect change (TT: 0.90, moderate effect vs. HFT: 0.39, small effect) for TT participants (Table 2). Figure 4 shows the CMJ height (cm) results before and after the intervention.

### 20 m change of direction test

No significant group by time interaction was observed for the 20 m COD test. Furthermore, planned comparisons within groups revealed no significant changes (TT: −0.04 ± 0.12 s; HFT: −0.02 ± 0.27 s). Both groups only showed a trivial magnitude of effect following the intervention (Table 2).

### Maximal voluntary isometric contraction (knee extension)

**Figure 5:**
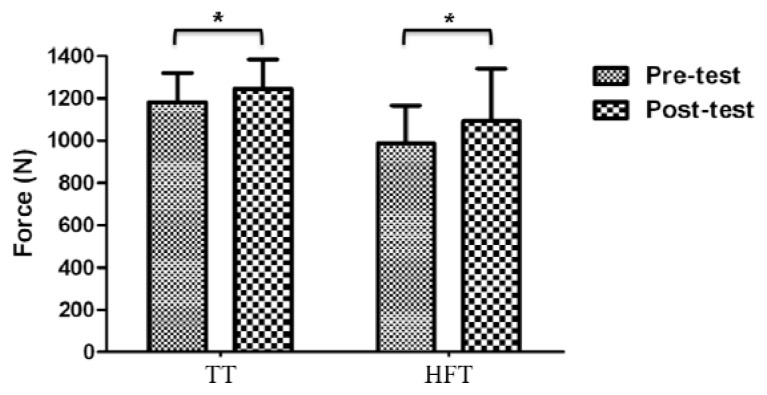
Pre- and post-training differences in maximal voluntary isometric contraction of knee extension. No significant group by time interaction (p > 0.05). *Significant planned comparisons within-group differences (p<0.05)

There was no significant group by time interaction observed in the MVIC test. However, planned comparisons within group analysis showed significant changes for the TT (62.87 ± 79.71 N, p=0.026) and HFT group (106.56 ± 121.63 N, p=0.011) with a greater magnitude of effect change for HFT participants (Table 2) (TT: 0.45, small effect; HFT: 1.02, moderate effect). Figure 5 shows the MVIC before and after the intervention.

## Discussion

This study aimed to assess the effect of six weeks of resistance training performed at the loads that maximized power using traditional training with a half squat (TT) versus training using a horizontal flywheel with a front step (HFT). Specifically, the study sought to understand if there were different adaptations in common sport-related tasks when using either the aforementioned traditional mode of power training versus power training that could be considered “plane specific”. Despite previous research demonstrating positive adaptations to traditional power training in the vertical plane (Cormie et al., 2007; Harris et al., 2008a; Newton et al., 2006; Ronnestad et al., 2008; Wilson et al., 1993), there has been little investigation on power training in the horizontal plane. We hypothesized that the TT and HFT would differ in their magnitude of improvement for each sport-related task based on the theory of specificity. However, in the current study a group by time interaction was only observed in the CMJH that demonstrated the TT group significantly improved the CMJH over the HFT group. Further planned comparisons demonstrated within-group improvements in the 10 m sprint and MVIC while only the TT group demonstrated a significant within-group improvement in 20 m sprint performance. The findings of this study do not provide support to previous suggestions that horizontal training will better translate to horizontal sport-related tasks such as sprinting and changing direction.

Multi-joint strength training is considered relevant to improve sprinting as sprinting requires powerful extensions of the hip, knee and ankle joints (Delecluse, 1997). However, a majority of studies using multi-joint training exercises are performed bilaterally in the frontal plane (McBride et al., 2002; Nimphius et al., 2012). Young (2006) suggested that the poor transfer of power training to tasks such as sprinting could be related to a lack of movement specificity. In this regard, one may expect a significantly greater 10 m sprint time in the HFT group, however, there was no significant group by time interaction observed, and the planned comparison revealed that both HFT (p=0.01; d=1.19) and TT (p=0.05; d=0.91) groups improved 10 m sprint performance with a moderate effect. Furthermore, planned comparisons revealed that only the TT group demonstrated significant improvements in 20 m sprint performance (TT: p=0.04; d=0.36 vs. HFT: p=0.23; d=0.11). Thus, the current study cannot currently support the statement that the more specific training method (HFT) transferred significantly better to sprint performance over TT. Such findings are in agreement with results of previous studies on traditional power training at Pmax that indicated significant improvements occurring at 10 m and 30 m distances in well-trained rugby athletes after 7 weeks of training (Harris et al., 2008a). The novelty of the current investigation is the comparison of TT to the “plane specific” power training of the HFT group. However, more research is necessary to investigate if the novel training method (HFT) can produce significant changes over beyond short-term interventions.

The only sport-related task that demonstrated a significant group by time interaction was CMJH. In the current study an improvement in CMJH of 14% (p = 0.001; d = 0.90) occurred following TT, which is in line with the results of a similar study using loads that maximized power (Cormie et al., 2007). Additionally, Wilson et al. (1993) reported a significant improvement in jump ability (17%) after a similar training program (10 weeks). The same argument about specificity of training for CMJH can be used to explain the significantly greater improvement in CMJH following TT over HFT. Many reasons could explain why the same level of specificity transfer to the “horizontal plane” movements such as sprinting or COD did not occur in the HFT group. For instance, the HFT method of loading may not allow for additional adaptations to occur through the entire kinetic chain since the loading for HFT is applied with a line of action through the trunk instead of through the trunk and legs. As a result, the direction of force through the stance phase in sprinting (Ross et al., 2001) or jumping is not as effectively replicated even though the apparent direction of travel during HFT training is visually perceived to be more “plane specific”. However, such a hypothesis as to why the adaptations to TT and HFT are different would require additional research with respect to neuromuscular adaptations to each type of training or variations in the loading used during training.

Despite the aforementioned improvements in sprint ability, the current study did not observe significant changes in COD. Some authors have shown a poor correlation between multi-joint leg extensor strength and power with COD in physically active men (Jones et al., 2009; Marcovic, 2007) that may explain the current findings. However, numerous studies provide better evidence than correlational studies for determinants of performance. Nimphius et al. (2012) demonstrated that 16 weeks of training with a strength phase preceding a power phase elicited significant changes in COD performance. The difference in the findings between the current study and that of Nimphius et al. (2012) is multifactorial, but may mostly be due to the shorter length (6 weeks) of the training program applied in the current research; another reason could be that the focus was laid on power training only. Previous research has shown that strength significantly explains changes in COD performance (Nimphius et al., 2012), while CMJH (or power) has not explained a large amount of the variance in COD performance at any phase of training (Jones et al., 2009; Marcovic, 2007; Nimphius et al., 2010). Therefore, the focus on power development using the Pmax load in the current study may not have been the best type and magnitude of stimulus required to elicit changes in COD performance. Furthermore, the lighter loads utilized during this study for power training may not provide the magnitude of eccentric stimulus that has been shown to be important for COD ability (Spiteri et al., 2014). Therefore, power training at the Pmax load, even when performed in a more “specific plane” of movement did not appear to improve COD performance. Additionally, future studies intending to use power training to improve COD ability should consider that previous research demonstrated that only higher relative maximal loads (80% in comparison to 30%) were able to significantly improve COD performance (McBride et al., 2002).

Numerous studies have reported an improvement in muscular strength following a resistance training program and therefore, the results of the current study with both groups demonstrating a significant improvement in knee extension MVIC are not surprising. Although, there was no significant group by time interaction, HFT demonstrated a moderate effect change in knee extension MVIC (p = 0.01; d = 1.02), whereas TT only resulted in a small magnitude of change (p=0.03; d = 0.45). The front step group may have benefited from the novel stimulus of the flywheel inertial device (Chiu et al., 2006) allowing for the slightly greater magnitude of improvement in strength of the quadriceps, as assessed by MVIC during the leg extension. However, the authors note that it has been suggested that working at a lower intensity (as when training at Pmax) is not a stimulus significant enough to maintain or improve strength and therefore, a mixed method approach of maximal strength training and power training is recommended (Haff and Nimphius, 2012). The six weeks of power training may have allowed for some improvement in these participants, but it would be expected this could not be sustained longitudinally without the development or inclusion of loading to improve maximal strength (Haff and Nimphius, 2012).

The direction of loading provided by TT versus HFT was clearly different and as a result, the absolute load used for training differed. The difference in absolute loading between the interventions (and therefore total work performed) is the main limitation of the current study. The primary purpose was to evaluate the effect of training at the load that maximized power for each respective group but it is acknowledged that total work could affect the findings. In addition, this study only applied six weeks of training and therefore, little is known about the long-term efficacy of horizontal inertial flywheel training. An additional limitation of the current study is the relatively low number of subjects tested in each group. Despite these limitations, it may be concluded that different modes of exercises (traditional training versus horizontal flywheel training), both using their respective Pmax load over six weeks, elicited different responses for each sport-related task. Future studies should investigate whether a greater volume, length or combination of training would result in different magnitudes of improvement.

## Figures and Tables

**Table 1:** Participants’ descriptive characteristics (mean ± SD)

Group	Age (years)	Body Height (cm)	Body Mass (kg)	BMI (kg·m^−2^)
TT	23 ± 3	76.51 ± 7.46	177.24 ± 4.61	24.36 ± 2.26
HFT	22 ± 2	77.47 ± 8.02	176.63 ± 2.27	24.81 ± 2.25

TT = traditional training using a half squat on a smith machine; HFT = horizontal flywheel training using a front step with an inertial flywheel device.

No significant difference between groups (p > 0.05)

**Table 2:** Within group planned comparisons pre- and post-training in traditional training and horizontal flywheel training groups for common sport-related tasks

	10 m sprint		20 m sprint		20 m COD		CMJH		MVIC	
Grou	*d*	*p*	*d*	*p*	*d*	*p*	*d*	*p*	*d*	*p*
TT	0.91	.048	0.36	.040	−0.08	.267	−0.90	.001	−0.45	.026
HFT	1.19	.011	0.11	.229	0.04	.871	−0.39	.050	−1.02	.011

TT = traditional training using a half squat on a smith machine; HFT = horizontal training using a front step with an inertial flywheel device; COD = change of direction; CMJH = countermovement jump height; MVIC = maximal voluntary isometric contraction; Cohen’ d (d) interpretation: <0.35 = trivial; 0.35–0.80 = small; 0.80–1.50 = moderate; ≥1.50 = large.

## References

[b1-jhk-47-155] Baker D (2001a). A series of studies on the training of high-intensity muscle power in rugby league football players. J Strength Cond Res.

[b2-jhk-47-155] Baker D (2001c). Acute and long-term power responses to power training: Observations on the training of an elite power athlete. Strength Cond J.

[b3-jhk-47-155] Baker D (2001b). Comparison of upper-body strength and power between professional and college-aged rugby league players. J Strength Cond Res.

[b4-jhk-47-155] Bemben MG, Rohrs DM, Bemben DA, Ware J (1991). Effect of resistance training on upper body strength, power and performance. J Appl Sport Sci Res.

[b5-jhk-47-155] Blazevich AJ, Gill N, Newton RU (2001). Reliability and validity of two isometric squat tests. J Strength Cond Res.

[b6-jhk-47-155] Chiu LZF, Salem GJ (2006). Comparison of joint kinetics during free weight and flywheel resistance exercise. J Strength Cond Res.

[b7-jhk-47-155] Cohen J (1998). Statistical Power Analysis for the Behavioral Sciences.

[b8-jhk-47-155] Cormie P, McCaulley GO, Triplett NT, Mcbride JM (2007). Optimal Loading for Maximal Power Output during Lower-Body Resistance Exercises. Med Sci Sports Exerc.

[b9-jhk-47-155] Cormie P, McGuigan MR, Newton RU (2011). Developing maximal neuromuscular power Part 2 – Training considerations for improving maximal power production. Sports Med.

[b10-jhk-47-155] Craig CL, Marshall AL, Sjöström M, Bauman AE, Booth ML, Ainsworth BE, Pratt M, Ekelund U, Yngve A, Sallis JF, Oja P (2003). International physical activity questionnaire: 12-country reliability and validity. Med Sci Sports Exerc.

[b11-jhk-47-155] Delecluse C (1997). Influence of strength training on sprint running performance. Current findings and implications for training. Sports Med.

[b12-jhk-47-155] Garber CE, Blissmer B, Deschenes MR, Franklin BA, Lamonte MJ, Lee IM, Nieman DC, Swain DP (2011). Quantity and quality of exercise for developing and maintaining cardiorespiratory, musculoskeletal, and neuromotor fitness in apparently healthy adults: guidance for prescribing exercise. Med Sci Sports Exerc.

[b13-jhk-47-155] Haff GG, Nimphius S (2012). Training principles for power. Strength Cond J.

[b14-jhk-47-155] Haff GG, Whitley A, Potteiger JA (2001). A brief review: Explosive exercises and sports performance. Strength Cond J.

[b15-jhk-47-155] Harris NK, Cronin JB, Hopkins WG, Hansen KT (2008b). Relationship between sprint times and the strength/power outputs of a machine squat-jump. J Strength Cond Res.

[b16-jhk-47-155] Harris NK, Cronin JB, Hopkins WG, Hansen KT (2008a). Squat jump training at maximal power loads vs. heavy loads: effect on sprint ability. J Strength Cond Res.

[b17-jhk-47-155] Hopkins WG (2005). Competitive performance of elite track-and-field athletes: variability and smallest worthwhile enhancements. Sport Science.

[b18-jhk-47-155] Jones P, Bampouras TM, Marrin K (2009). An investigation into the physical determinants of change of direction speed. J Sports Med Phys Fitness.

[b19-jhk-47-155] Kawamori N, Crum AJ, Blumert PA, Kulik JR, Childers JT, Wood JA, Stone MH, Haff GG (2005). Influence of different relative intensities on power output during the hang power clean: identification of the optimal load. J Strength Cond Res.

[b20-jhk-47-155] Kawamori N, Haff GG (2004). The optimal training load for the development of muscular power. J Strength Cond Res.

[b21-jhk-47-155] Little T, Williams AG (2005). Specificity of acceleration, maximum speed, and agility in professional soccer players. J Strength Cond Res.

[b22-jhk-47-155] Marcovic G (2007). Poor relationship between strength and power qualities and agility performance. J Sports Med Phys Fitness.

[b23-jhk-47-155] Lohnes CA, Fry AC, Schilling BK, Weiss L (2007). Kinetic Comparison Between Various Resistance Settings on the Versa-Pulley™ Training System. Medicine & Science in Sports & Exercise.

[b24-jhk-47-155] Mayhew JL, Johns RA, Ware JS, Bemben MG, Bemben DA (1992). Changes in absolute upper body power following resistance training in college males. J Appl Sport Sci Res.

[b25-jhk-47-155] McBride JM, Triplett-McBride T, Davie A, Newton RU (1999). A comparison of strength and power characteristics between power lifters, Olympic lifters, and sprinters. J Strength Cond Res.

[b26-jhk-47-155] McBride JM, Triplett-McBride T, Davie A, Newton RU (2002). The effect of heavy- vs. light-load jump squats on the development of strength, power, and speed. J Strength Cond Res.

[b27-jhk-47-155] Moss BM, Refsnes PE, Abildgaard A, Nicolaysen K, Jensen J (1997). Effects of maximal effort strength training with different loads on dynamic strength, cross-sectional area, load-power and load-velocity relationships. Eur J Appl Physiol Occup Physiol.

[b28-jhk-47-155] Newton RU, Cormie P, Kraemer WF, Hoffman J (2012). Power training, National Strength and Condition Association. NSCA’s guide to program design.

[b29-jhk-47-155] Newton RU, Dugan E (2002). Application of strength diagnosis. Strength Cond J.

[b30-jhk-47-155] Newton RU, Kraemer WJ, Hakkinen K, Humphries B, Murphy AJ (1996). Kinematics, kinetics, and muscle activation during explosive upper body movements. J Appl Biomech.

[b31-jhk-47-155] Newton RU, Kraemer WK (1994). Developing explosive muscular power: Implications for a mixed methods training strategy. Strength Cond J.

[b32-jhk-47-155] Newton RU, Rogers RA, Volek JS, Hakkinen K, Kraemer WJ (2006). Four weeks of optimal load ballistic resistance training at the end of season attenuates declining jump performance of women volleyball players. J Strength Cond Res.

[b33-jhk-47-155] Nimphius S, McGuigan MR, Newton RU (2010). Relationship between strength, power, speed, and change of direction performance of female softball players. J Strength Cond Res.

[b34-jhk-47-155] Nimphius S, McGuigan MR, Newton RU (2012). Changes in muscle architecture and performance during a competitive season in female softball players. J Strength Cond Res.

[b35-jhk-47-155] Norrbrand L, Pozzo M, Tesch PA (2010). Flywheel resistance training calls for greater eccentric muscle activation than weight training. Eur J Appl Physiol.

[b36-jhk-47-155] Rhea MR (2004). Determining the magnitude of treatment effects in strength training research through the use of the effect size. J Strength Cond Res.

[b37-jhk-47-155] Ronnestad BR, Kvamme NH, Sunde A, Raastad T (2008). Short-term effects of strength and plyometric training on sprint and jump performance in professional soccer players. J Strength Cond Res.

[b38-jhk-47-155] Ross A, Leveritt M, Riek S (2001). Neural influences on sprint running: training adaptations and acute responses. Sports Med.

[b39-jhk-47-155] Sleivert G, Taingahue M (2004). The relationship between maximal jump-squat power and sprint acceleration in athletes. Eur J Appl Physiol.

[b40-jhk-47-155] Spiteri T, Nimphius S, Hart NH, Specos C, Sheppard JM, Newton RU (2014). The contribution of strength characteristics to change of direction and agility performance in female basketball athletes. J Strength Cond Res.

[b41-jhk-47-155] Stone MH, O’Bryant HS, Mccoy L, Coglianese R, Lehmkuhl M, Shilling B (2003). Power and maximum strength relationships during performance of dynamic and static weighted jumps. J Strength Cond Res.

[b42-jhk-47-155] Turner AP, Unholz CN, Potts N, Coleman SG (2012). Peak power, force, and velocity during jump squats in professional rugby players. J Strength Cond Res.

[b43-jhk-47-155] Wilson GJ, Newton RU, Humphries BJ, Murphy AJ (1993). Maximising the performance gains from resistance training: the optimal training modality.

[b44-jhk-47-155] Winchester JB, Erickson TM, Blaak JB, McBride JM (2005). Changes in bar-path kinematics and kinetics after power-clean training. J Strength Cond Res.

[b45-jhk-47-155] Young WB (2006). Transfer of Strength and Power Training to Sports Performance. Int J Sports Physiol Perform.

[b46-jhk-47-155] Zink AJ, Perry AC, Robertson BL, Roach KE, Signorile JF (2006). Peak power, ground reaction forces, and velocity during the squat exercise performed at different loads. J Strength Cond Res.

